# Integrating collaborative place-based health promotion coalitions into existing health system structures: the experience from one Australian health coalition

**DOI:** 10.5334/ijic.2012

**Published:** 2015-12-15

**Authors:** Carolyn Ehrlich, Elizabeth Kendall

**Affiliations:** Centre of National Research on Disability and Rehabilitation, Menzies Health Institute Queensland, Griffith University, Logan Campus, Meadowbrook, QLD 4131, Australia; Centre of National Research on Disability and Rehabilitation, Menzies Health Institute Queensland, Griffith University, Logan Campus, Meadowbrook, QLD 4131, Australia

**Keywords:** place-based initiatives, partnerships, social systems, health care systems, embedding interventions, integrating care

## Abstract

**Background:**

Increasingly, place-based collaborative partnerships are being implemented to develop the capacity of communities to build supportive environments and improve population health outcomes. These place-based initiatives require cooperative and coordinated responses that can exist within social systems and integrate multiple responses. However, the dynamic interplay between co-existing systems and new ways of working makes implementation outcomes unpredictable.

**Method:**

We interviewed eight programme leaders, three programme teams and two advisory groups to explore the capacity of one social system to implement and normalise a collaborative integrated place-based health promotion initiative in the Logan and Beaudesert area in South East Queensland, Australia. The construct of capacity as defined in the General Theory of Implementation was used to develop a coding framework. Data were then placed into conceptually coherent groupings according to this framework until all data could be accounted for.

**Results:**

Four themes defined capacity for implementation of a collaborative and integrated response; namely, the ability to (1) traverse a nested and contradictory social landscape, (2) be a responsive and ‘good’ community partner, (3) establish the scaffolding required to work ‘in place’; and (4) build a shared meaning and engender trust. Overall, we found that the capacity of the system to embed a place-based health promotion initiative was severely limited by the absence of these features.

**Conclusion:**

Conflict, disruption and constant change within the context into which the place-based collaborative partnership was being implemented meant that existing relationships were constantly undermined and the capacity of the partners to develop trust-based coherent partnerships was constantly diminished. To enhance the likelihood that collaborative and integrated place-based health promotion initiatives will become established ways of working, an agreed, meaningful and clearly articulated vision and identity are required; goals must be prioritised and negotiated; and sustainable resourcing must be assured.

## Introduction

Taking action to improve population health outcomes in areas of high deprivation has become a global social and economic imperative [1–3]. Many contextual factors impact on health care service delivery, and interventions aimed at achieving improved population health outcomes need to be implemented within existing contexts [4]. Health care systems consist of multiple service providers, professional or disciplinary groups, and sectors [5] and have been built on differentiation, specialisation and segmentation. Hence, siloed mindsets that result in fragmented, disjointed care have been created [6]. This approach is considered to be inefficient, costly and of sub-optimal quality [7]. In the absence of integrated management hierarchies, organisations need to voluntarily cooperate and collaborate with each other to achieve integrated service delivery [5]. Thus, locally developed collaborative partnerships or coalitions are increasingly being established in an attempt to integrate service delivery in ways that build health-promoting communities [8–10].

Over the past decade, most governments in developed countries have introduced geographically targeted projects [11] that promote ‘whole-of-community’ (i.e. integrated) planning to achieve desired health outcomes [12]. In Queensland, Australia, the government funded a number of geographically defined (i.e. place-based) integrated care initiatives between 2006 and 2012 to address the increasing impact of chronic diseases and the associated pressure on health care provision. In South East Queensland, one of the place-based initiatives was funded in the geographical areas of Logan and Beaudesert. This geographical area is recognised as being socially disadvantaged and culturally diverse. These initiatives were one component of a comprehensive chronic disease strategy [13]. The focus of the initiatives was to build new and collaborative ways of working and to produce local, integrated models of service delivery that spanned the health care continuum. At a macrolevel, the initiatives were focused on achieving organisational integration (i.e. strategic inter-agency alliances and jointly managed programmes to fit the needs of people across the continuum) by using strategies that link similar levels of care with each other (i.e. improving overall health using peer-based and cross-sectorial collaborative approaches) [6,7].

If these new initiatives were to be successful, the existing social structures and norms (i.e. socially constructed way of working) into which they are being implemented needed to have the capacity to deliver a cooperative and coordinated response [14–16]. Capacity to achieve this connectivity across micro-, meso- and macrolevels requires coherence among existing values, methods of organising service delivery and components of the clinical delivery systems [6,7]. This coherent way of working must become embedded within existing contexts if these new initiatives are to be sustained across time and reproduced in new spaces [6,17,18]. Successfully implementing integrated methods of service delivery also means that the existing social system may need to change, necessitating capacity for modification [16]. Unless the existing social system has the capacity to modify and engage in collaborative partnerships, it will be extremely challenging to successfully introduce an integrated whole-of-community health promotion initiative.

Evidence suggests that if whole-of-community ways of working are to become normatively integrated (i.e. embedded) in practice, they need to ‘fit’ with the routines and practices of existing social systems [17,19]. These social systems comprise interacting networks of relational pathways that unfold over time and either facilitate or inhibit the uptake of new ways of working [15,16]. Within existing relational networks, different organisational and professional cultures (i.e. roles, values, experiences, expectations and power distributions) influence the ways in which health promotion activities need to be integrated [20].

According to some theorists [15], institutional life in modern society is in a constant state of organisation and re-organisation and social order is defined by the way in which individuals or collective groups interact with knowledge about one another, and understand the purposes, relationships, power and rules of the field in which they are operating. When new collaborative and integrated initiatives are implemented, the existing social order will be re-organised and a dynamic interplay between the stakeholders and existing resources will occur. This dynamic interplay shapes the capacity of the system to accommodate and embed new practices, thereby influencing implementation in ways that can make outcomes unpredictable [16].

Social systems that have the ability and resources (i.e. capacity) to act collectively are more likely to successfully embed complex interventions [16]. Although embedding complex interventions in practice is important, there has been a dearth of theory available to guide the implementation processes. One recent theory that contributes to understanding the implementation process is May’s General Theory of Implementation [16]. Central to the theory is the notion that new practices are implemented within social systems. Several socio-structural resources are associated with the capacity of the social system to embed practices, namely the social norms and roles contained within networks, and the material and cognitive resources available to network members [16]. The knowledge and understanding held by existing and new entrants to the field contributes to the unpredictability of the system and implementation efforts will be affected by who does and who does not benefit in the new order [15].

For the Queensland collaborative and integrated health promotion initiatives to become embedded, the existing health and social care systems would require sufficient capacity to work in integrated ways. Our study used qualitative methods to explore the capacity of one of these initiatives, the Logan-Beaudesert Health Coalition. We used May’s [16] concept of socio-structural resources to explore capacity within existing social systems, that is, we reasoned that the capacity to adopt an integrated health promotion initiative would be dependent on understanding the: (1) rules and norms of the existing social order, (2) roles and relationships among individuals and collectives, (3) individual and collective knowledge about the social order and (4) distribution and allocation of material resources available to the actors. The purpose of this paper is to report the results of a qualitative study that explored the capacity of one health care system to implement a place-based integrated health promotion initiative within usual service delivery processes.

## Methods

### Setting

The purpose of the collaborative integrated health promotion initiative, the Logan Beaudesert Health Coalition (hereafter referred to as the Coalition), was to develop new ways of addressing the increasing burden of chronic disease by: (a) engaging a range of providers and sectors; (b) integrating a range of approaches to disease management; (c) shifting the focus of the health care system towards illness prevention and health promotion; (d) addressing issues of equity and access; (e) applying a life course approach to health across the continuum; and (f) focusing on all health determinants. Although the government funded and implemented several place-based initiatives throughout Queensland, the initiative on which this research was based occurred in an outer suburban setting characterised by low socio-economic status, high disease rates and diverse ethnicity.

The Coalition integrated six programme areas under the governance of one overarching body. The programme areas focused on: (1) early years (i.e. birth to 8 years), (2) chronic condition management, (3) multicultural health, (4) tertiary and primary care integration, (5) information sharing and management, and (6) health promotion. The early years and chronic condition programmes received the greatest amount of funding and employed multiple staff, while other programme areas each employed a single staff member to coordinate activity. Both the early years and chronic condition programme areas were initially co-located in partner organisations. However, the early years programme was subsequently located within the State Health Department when appropriate accommodation became available. Four of the six programme areas (i.e. not information management and the chronic condition programme areas) established a broad group of interested stakeholders to act as an advisory body during the implementation process. The overarching governing body was established after the programme areas had commenced implementation and comprised eight members of key organisations. Members were recruited to oversee programme implementation and plan new interventions. A project leader was employed to manage the entire initiative. This research was conducted once the governing body had been established for 12 months to ensure that all programmes had progressed beyond the early stages of implementation. Approvals from the relevant Human Research Ethics Committees were obtained.

### Data collection

To determine capacity for implementing integrated health promoting care, each programme leader was interviewed individually. However, given the importance of social networks to implementation theory, teams and advisory groups were also interviewed as collectives. All interviews were conducted face-to-face, in a place and at a time that was convenient for each participant, or group of participants. A semi-structured interview guide was used to explore participants’ experiences of acting collectively during the implementation process. Questions included: How have the principles and values of the Coalition been enacted during implementation of individual programmes? How do you foster links with other groups? What have been some of the major challenges for your programme? What will you do to try and sustain involvement over time? How well do you think the programme has fitted with the broader environment?

### Participants

Eight programme leaders (PL), three programme teams (PT) and two advisory groups (AG) were interviewed. Table 1 outlines the intervention area and the individuals or groups who were included in the interviews.

### Data analysis

Each interview was transcribed verbatim and checked for accuracy. Both authors openly and independently read each transcript. Because of the interpretive nature of the research and the richness of the data, in-depth qualitative analysis of data was undertaken using the following steps:

1. Using NVivo 10^TM^ as a data management tool, transcripts were coded against a framework derived from dimensions of the capacity construct defined in General Theory of Implementation [16], that is social norms, social roles, and material and cognitive resources.
2. The initial coding was then re-examined by asking the question, ‘What is this specifically?’ (i.e. second-level coding) [21].
3. Data were then placed into groups that seemed to belong together conceptually [22,23]. Both authors interrogated this conceptual coding to determine the meaning of clusters until all data could be accounted for [22,24]. The results are presented in the next section and are organised according to the four elements of capacity as defined by May [16]. To maintain anonymity of participants, quotes are attributed to either programme leaders (PL), programme teams (PT) or advisory groups (AG).

## Results

Four themes reflected the capacity of the existing health care social system to integrate and embed a purpose-specific, locally developed health Coalition. The four themes were: (1) traversing a nested and contradictory social landscape, (2) being a responsive and ‘good’ community partner, (3) the scaffolding required to work within ‘place’ and (4) building shared meaning and engendering trust. Interwoven throughout each theme was the importance of a shared and meaningful identity. Without an agreed and articulated identity, the capacity for the Coalition to integrate within the existing health care social system was restricted.

### Traversing a nested and contradictory social landscape

The Coalition had ambitious goals:

“… what we’re trying to do is address gaps in service delivery, so looking at enhancing existing delivery, developing new services and be[ing] innovative, but also looking at creating a ‘system shift’ in the way we address health in the [geographical] area, so looking at advocacy and policy and capacity building”. (PL)

This vague and broad purpose established a normative culture for the Coalition of being innovative, challenging boundaries and finding new ways of working across artificial divides. However, the ambition to create innovative change regularly opposed normative ways of working within the broader social landscape, which meant that participants had to deal with:

“… some internal battles and turf issues [that] are still there between some organisations. I think that [it] is decreasing. I think we’re seeing less of that but in some areas it’s still quite strong between one organisation in particular there are some turf battles”. (PL)

The broader social landscape was characterised by hierarchical decision-making and top-down developmental processes. The acute care focus of the funding organisation overshadowed the Coalition focus on illness prevention and health promotion, “You’re talking to people in [State Government Department] who very much focus on the acute sector; on the hospitals” (PL). Consequently, some participants felt powerless to implement new or different ways of working. When conflict arose, participants sometimes responded by disengaging, “Then we had a problem with [another department] but we just backed off with that, which was sad” (PL). Thus, the broader hierarchical norms stifled creativity, leaving little room for innovation.

Continual change and reform in the funding organisation created unpredictability, which unsettled the Coalition partners, “We’ve had several restructures with [State Government Department], there’s been local government restructures, there’s another [State Government Department] restructure or reform that’s coming up … [it] made my work harder” (PL). Changes were believed to be unrelenting and expensive,

“That [change in service provision by another department] was very costly. That tells you how individuals in here - people in here have changed. Even the management of the hospital has changed. That is a costly thing”. (PL)

Unrelenting change meant that the Coalition was unable to articulate a tangible vision, which impacted negatively on integration:

“that raises the importance of ‘what’s our vision as a group - what are we trying to achieve here?’ If the vision across the programmes is different, then you’re going to have huge integration problems anyway. So it is about our shared visions”. (PL)

Despite the importance of a shared identity, “… it’s really clear that our [Coalition] identity and our professional identity is core” (PL), the Coalition was unable to attain its own identity, “I don’t think there’s a huge amount of awareness about exactly who we are” (PL). Thus, the identity of the larger and more influential funding organisation prevailed, leading to inherent confusion and uncertainty about the Coalition’s role. In turn, there was a sense that the Coalition lacked stability, clarity and durability.

Participants believed that the politically oriented context in which they were situated was incongruent with the underlying philosophy of a place-based initiative. They believed that the Coalition had been imposed rather than being an organic response to community identified needs. Some local organisations who had a long history of delivering community-responsive services struggled to articulate how or why they should integrate with the Coalition “I think probably we [existing community organisation] need to work more as a group to see how we fit in in there [with the Coalition]. I think the programmes here [existing organisation] fit but it’s how we fit as a group within the coalition” (PL). The incongruence between bureaucratic and community needs left participants unclear about how to adopt a place-based philosophy - a perception that was compounded by the restrictive and continually changing environment. It also meant that although participants held a place-based health promotion philosophy, they had little influence in the broader health care context and were expected to conform to the external forces that were directing the speed and nature of change.

### Multiple conflicting roles - being a ‘responsive’ and ‘good’ partner

The grassroots philosophical approach of staff was contained and constrained within a highly politicised and hierarchical health system environment, which created a dilemma for participants. Participants needed to listen and respond to community goals. However, there was a high level of identified need in a community that was diverse and transient, “… There’s so much need out there” (PL), “… it is such a diverse community that we’re working in” (PL), “… we’ve got a huge turnover of people coming through [the area]” (PT). Simultaneously, participants had to work within policy constraints:

“… we need to line it [current service provision work] up with current government priorities so we continually [need to give our partners] the message that we are not doing this [whatever the community believes is a priority] not because the community doesn’t find it an important issue, [but because of current policy restrictions]”. (AG)

One response to the diversity, transience and complexity was to take creative action and to think laterally in ways that

“… facilitated change [by] looking at where the other gaps are. This year we’re trying to implement a research and innovation programme, which will [occur because of] some funding that the board can access to actually do some of the new work. To help that change as well we’re looking at - and once again this is something that hasn’t been done particularly well in the past but we’re about to embark on a new process around decision making and planning”. (PL)

“So, I would like to see a lot more breaking-down of barriers, with people taking a little bit of a lateral view of things and maybe having pilot projects of some of the out-of-left-field type ideas that we have”. (AG)

However, in a bureaucratic context, there was little room for creativity. Even within the Coalition, there were “… some ingrained cultures about how we do things and not much shifting is going on” (PL), which created internal tension and presented as role confusion.

Participants needed to develop and maintain partnerships at multiple levels (i.e. with individuals, communities, programme areas, groups and organisations). Partners were highly regarded if they could assist the Coalition to meet the mandate. Thus, the focus was on the Coalition rather than on the needs of partners. It was important to know “[how] they [other organisations] perceive what the Coalition is doing and are we threatening?” (PL). Participants needed to ensure that partners and stakeholders became familiar with and endorsed the work of the Coalition, while simultaneously ensuring that partners did not feel that the Coalition was encroaching on their areas of expertise. Thus, they needed highly developed social skills to be able to recognise and interpret their environment and mobilise people to engage in collective action.

It was difficult to identify appropriate partners and then be clear about how they would work together “We can always work with the converted, all those who [are of a] similar ilk, but it’s the ‘tough nuts’ we need to work with … people who work with a different perspective …” (PT). This dilemma placed participants in the confusing role of having to build partnerships that were not naturally easy or comfortable when they were more attracted to those with similar beliefs and values. Thus, there was evidence that participants did not always have the capacity to engage with partners in ways that assisted sense-making about the underlying purpose, rules and relationships of the dominant organisation.

An important role of participants was to develop the capacity of their community partners “… we build capacity and we move on” (PL). Capacity building was sometimes referred to as community development and was usually designed to create either a shift in current practice or to build sustainability. To enhance capacity participants relied on skill-building strategies, “we support a learning environment or a learning community” (PL). Paradoxically, therefore, participants had to impart new skills in ways that fit with and valued ingrained community methods. Due to inherent role confusion, bureaucracy frequently took precedence over the needs of community partners, “when we first started out we were somewhat egocentric in that … somehow it was all about us, and [we assumed] that people were going to give us their time and their effort and that it was just all about our work” (PL). Thus, partnerships were sometimes short-lived because Coalition members assumed dominance, expertise and responsibility for building the capacity of ‘junior’ partners. This approach threatened partnership durability and reduced overall capacity for integration.

### The scaffolding required to work within ‘place’

Finite resources meant that participants relied on partner organisations to deliver some aspects of the Coalition’s work, “we can’t be everywhere for everyone … We’ve got to look at where there are no services, or there are limited services [that could be] enhanced” (PT). Although participants were aware that they needed to work in a collaborative space, they needed strategies and agreed processes to deliver their mandate, especially with regard to community engagement, “Collaboration and coordination, I think everyone’s aware of that and are largely working in that manner. Community engagement is probably one of the ones that isn’t occurring as much” (PL). Processes were required to achieve cultural shifts and to partner with communities:

“Yeah, so the hoarding of information … is very destructive in especially a partnership organisation like this. So I think that’s a cultural shift that needs to happen for sure and there need to be processes around that to facilitate that process”. (PL)

These complex goals required participants to “focus [on] the project management side of things. So scheduling, documentation, communication - you know - managing that in a defined methodology. That’s really the crux of my process that I use” (PL). Participants also needed access to “… the right information, the research, the tools to bring about that change …” (PL), because “information bridges the gap between the [programme] areas and also across the Coalition as a whole” (PL). Thus, developing a shared understanding between the linkages and networks that existed between health promotion and other fields of health care action were critical to the success of the initiative.

To achieve their goals, participants also needed sustainable resources “… our needs are probably more based around human resources … there is only so much that one person can do” (PT). The financial vulnerability of the programmes was constantly reiterated, “… if the funding ceased today the programme would stop. I don’t think sustainability has been built in” (PL). Ironically, participants stated that they needed more resources and financial security to do their work effectively, but simultaneously encouraged their partners to continue their work despite a lack of resources and funding.

### Building shared meaning and engendering trust

Participants believed that shared knowledge about the history of the Coalition was important to developing shared understanding and trust. Some participants who had not been involved from the inception of the Coalition found it difficult to understand why some aspects of programmes existed “… I think when we started we missed a lot of ‘how everything came to be” (PT). They believed that some programmes were inconsistent with the nature of a place-based initiative, “Yes, I’m not sure to what extent the community informed the selection of the programmes …” (PT). “… I think some of the issues [being addressed by the programmes] were political, not because there was a need” (PL). Thus, for participants who had not been involved in the development of the Coalition, an historical perspective was important.

Multiple communication and engagement structures were used to inform partners, “We have our ‘Communication Framework’, which is new and we have our ‘Planning and Implementation Framework’ which is also new and that sits across everything” (PL). Although these structures had been internally created, other externally generated frameworks were also used to facilitate shared understanding:

“so the model which I’m using … is the [name of a social determinants of health framework] and using some of these processes to bring teams together… [and] to help us refine the information that’s out there into something that’s palatable for the board [Coalition governing body]”. (PL)

However, when frameworks and structures failed to create meaning and ways of belonging, participants needed to “… alter, make up new continua and conceptual frameworks to sit ourselves within in the Coalition and to diffuse any tensions …” (PT).

These frameworks needed to cross many artificial boundaries within the Coalition that mitigated trust “… people are coming from different backgrounds, different agencies, they have their own culture, their own language, so that language is still getting in the way in some areas …” (PL). Within the broader community “… there are a lot of issues which directly and indirectly relate to culture and language and religion [that] you need to deal with” (PL). Thus, participants needed to be culturally competent and able to work comfortably with diversity. They needed to simultaneously respect boundaries and find ways to cross them. Participants needed to ensure that their work was meaningful for their partners as well as for their own organization, “… but I think if we’re going to link and collaborate with them, it needs to be meaningful. We need to do if for the right reasons” (PL). However, a trusting relationship with partners relied on the ability to clearly communicate the purpose of the Coalition, but this remained elusive.

## Discussion

In this study, we used General Theory of Implementation to explore the capacity of an existing social system to integrate a place-based health-promoting service delivery health Coalition within existing care systems. We found that all four elements of capacity as defined in the General Theory of Implementation [16] were compromised. The rules and norms of the predominant partner impacted on the way in which Coalition members were able to interact with other partners. The roles of participants were not clearly defined, which meant that relationships between potential partners were vulnerable to mistrust, disharmony and conflict. In the absence of a clear and collectively derived vision or purpose, ways of operationalising the Coalition were open to individual interpretation, which added to the sense of disharmony and conflict. Finally, despite ambitious goals, resource distribution and the funding role of the predominant partner impacted on the capacity of the Coalition to become embedded within the existing care system.

### Rules and norms

Some researchers have argued that an ideal approach to integration is to achieve a balance between top-down (i.e. directives that encourage implementation, evaluation and sustainability) and bottom-up (i.e. participative leadership and engagement) approaches [20]. The Coalition was borne from policy directives (i.e. top-down), but was required to use participatory engagement strategies to achieve grass-roots engagement. Overwhelmingly, our findings indicated that the Coalition was operating in a broader system that was fluid, open to contest and in a constant state of change. Members were constantly making adjustments to their work in response to their perceptions of the politically driven changes that were occurring in the field [15]. Without the skill to translate rules and resources into productive partnerships, the capacity of the Coalition to become embedded (i.e. normatively integrated) in the existing social order was compromised [6,7,16]. Thus, the ability of the Coalition to establish a meaningful and well-recognised identity within the existing social system [16] was impeded.

### Roles and relationships

Ongoing disruptions within the Coalition and its complicated context meant that existing relationships were constantly undermined and new meanings about the nature of, and roles within, relationships had to be developed [15]. Collaborative partnerships that supported co-location of services and jointly managed programmes were fundamental to the integration work of the Coalition [7]. However, the contradictory social landscape made it difficult for Coalition workers to understand and then translate the rules and resources that underpinned partnerships in ways that encouraged potential partners to collaborate [15].

### Individual and collective knowledge about social order

The Coalition was situated within a complex and constantly changing hierarchical health system that often contradicted participants understanding of their role and mission. Constantly changing parameters meant that participants found it challenging to develop a shared meaning of ‘place’. Members of the Coalition were expected to work in collaboration with a broad network of stakeholders. However, the existing network of relationships was complex, which resulted in difficulty establishing the shared meaning and trust base that was required to build responsive partnerships. Overall, the system had limited capacity to integrate a place-based health promotion initiative into its standard way of operating.

Although whole-of-community approaches are implemented to benefit the community [25], this integrated health promotion initiative was imposed on the community within a highly politicised health system. Therefore, obtaining community cooperation was difficult because of the actions of the dominant funding partner [15]. Securing the cooperation of others was essential for the stability of the Coalition, but required well-developed capacity for successful engagement [15]. Engagement capacity was dependent on the ability of participants to access the information and knowledge that they needed to operationalise the Coalition [16]. However, in the constantly changing environment, the information and knowledge that was required to build trust, consolidate a coherent sense of ‘place’ and develop a clear and shared vision with community partners [26] was unable to be established.

## Conclusion

This research occurred during a particularly tumultuous period for health services in Queensland and in Australia. Funding models and geographical boundaries of health service districts, local government areas and federally funded primary care regions were in a continual state of flux. Thus, the extent to which these findings will be transferable to other contexts may be limited. Despite this limitation, we believe the research has exposed several recommendations for implementing collaborative and integrated place-based initiatives into practice.

Although it has been proposed that a mix of both bottom-up and top-down approaches to integration is required, we have identified that achieving a balance between these two approaches is challenging. We have identified that the ability of existing social systems to normalise these types of integrated approaches to health promotion is restricted when the socially constructed interactions between groups are inconsistent with the collective action required. To enhance the capacity for integration of these interventions there needs to be an agreed, meaningful and clearly articulated vision and identity. However, achieving this clarity of vision and identity is reliant on well-developed skills to accurately identify and then decisively act on the inherent rules and norms contained within the social landscape in ways that convince collaborators and potential partners to engage with these new ways of working.

Finally, capacity for integrated health promotion initiatives to become normalised across multiple sectors was likely to be enhanced with sustainable resourcing. In the context of this research, sustainable resourcing included both material and cognitive resources such as access to trusting and meaningful relationships, shared meaning, historically based contextual knowledge and appropriate levels of time and funding. True collaboration and cooperation were required.

## Figures and Tables

**Table 1.**
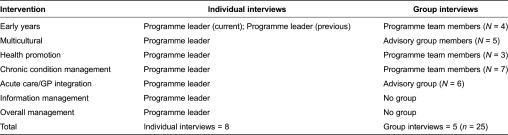
Participant details
